# A Dilated Residual Network for Turbine Blade ICT Image Artifact Removal

**DOI:** 10.3390/s23021028

**Published:** 2023-01-16

**Authors:** Rui Han, Fengying Zeng, Jing Li, Zhenwen Yao, Wenhua Guo, Jiyuan Zhao

**Affiliations:** 1 State Key Laboratory for Manufacturing Systems Engineering, Xi’an Jiaotong University, Xi’an 710049, China; 2 China Gas Turbine Establishment, Aero Engine Corporation of China, Chengdu 610500, China; 3 School of Automation, Beijing Information Science and Technology University, Beijing 100192, China

**Keywords:** Industrial Computed Tomography, turbine blade, convolution neural network, artifact removal

## Abstract

Artifacts are divergent strip artifacts or dark stripe artifacts in Industrial Computed Tomography (ICT) images due to large differences in density among the components of scanned objects, which can significantly distort the actual structure of scanned objects in ICT images. The presence of artifacts can seriously affect the practical application effectiveness of ICT in defect detection and dimensional measurement. In this paper, a series of convolution neural network models are designed and implemented based on preparing the ICT image artifact removal datasets. Our findings indicate that the RF (receptive field) and the spatial resolution of network can significantly impact the effectiveness of artifact removal. Therefore, we propose a dilated residual network for turbine blade ICT image artifact removal (DRAR), which enhances the RF of the network while maintaining spatial resolution with only a slight increase in computational load. Extensive experiments demonstrate that the DRAR achieves exceptional performance in artifact removal.

## 1. Introduction

**Figure 1:**
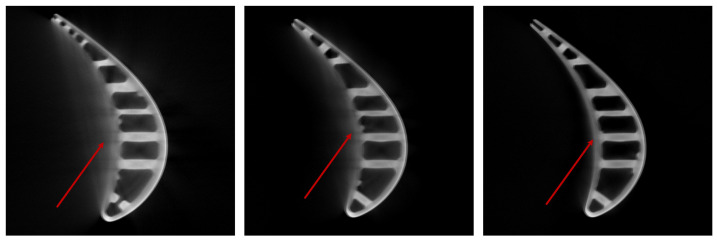
ICT images of turbine blades affected by different degrees of artifacts—the degree decreases from left to right. Severe artifacts have seriously affected the measurement of the wall thickness of blades in CT images.

ICT (Industrial Computed Tomography) has been widely used in defect detection [1,2,3,4,5], dimensional measurements [6,7], and geometric analysis [8,9], including in the aerospace field [5,10], vehicle manufacturing [11,12], additive manufacturing [3,5,8], etc. However, due to the influence of beam hardening and scattering during CT scanning and imaging, there are artifacts on the obtained cross-section images, as illustrated in Figure 1. The area indicated by the red arrow in Figure 1 is the area where an artifact exists, and the degree of artifact influence decreases from left to right. The existence of artifacts makes the image degenerate severely and reduces the accuracy of defect detection and dimensional measurement.

Metal artifacts are the most common phenomenon in the tomography of turbine blades, which are commonly seen in the scanning of objects with two or more kinds of constituent materials and large differences of density in materials. The metal artifacts are shown explicitly as random emission strip artifacts around the high-density materials. Metal artifacts reduce the contrast of ICT images and erode the real structure of the scanning object. As shown in Figure 1, the edge of the blade is completely covered by the artifacts, and the contours are all fuzzy; thus, it is challenging to achieve accurate contour extraction.

To reduce the impact of artifacts on CT applications, various artifact removal (AR) methods have been proposed. Among these methods, interpolation-based methods are the most common [13,14,15,16]. Interpolation-based methods first segment the metal regions by threshold segmentation and then use different interpolation methods to filter or denoise the adjacent data to correct the non-metal region. The performance of these methods is greatly affected by the segmentation quality [17], the effect of them is unstable, and they are mainly applicable to medical CT using X-ray [18]. Other common methods are iterative reconstruction (IR) methods [19,20,21]. These methods use the characteristic information of the object to establish the cost function and use an iterative method to approximate the true value to correct artifact information. IR methods demand a long running time and significant computing resources.

Thanks to the powerful learning and feature representation capability of convolutional neural networks (CNNs), the learning-based AR methods [22,23,24,25,26] have achieved far better performance than previous methods. Kida et al. [22] designed a deep convolutional neural network (DCNN) based on UNet for the correction of scattering artifacts and truncation artifacts to improve CBCT image quality. Harms et al. [23] proposed a residual block concept into a cycle-consistent adversarial network (cycle-GAN) framework (res-cycle GAN) for the correction of scattering pseudopacities. Xiao et al. [24] proposed a geometric artifact correction method based on a fully convolutional neural network. Zhu et al. [25] also proposed a GAN framework, using the U-Net structure as the generator to extract the CT image features with geometric artifacts. Busi et al. [26] introduced the 3D U-Net architecture into this field and achieved better performance. These demonstrate the effectiveness of CNNs applied to CT artifact removal.

At present, the learning-based methods have achieved the best and most stable performance. However, most of the existing methods take medical CT as the research object, and there is less research on the artifact removal of ICT images, especially turbine blade ICT images. Moreover, most of the previous network models seek to introduce new modules or blocks but ignore the main influencing factors that affect the performance of the model in the AR task. In addition, there is no uniform method for the preparation of AR datasets of ICT images at present. Based on the existing CNN method, this paper studies the main influencing factors in the model design for the ICT image artifact removal task to design an effective artifact removal model. The main works are as follows:

- We propose a method to prepare a dataset for turbine blade ICT images artifact removal. For any given slice image, the blade contour is fitted, and then the artifact information outside the contour is manually removed; then, the result is taken as ground truth.
- We design two different methods for AR to study the main influencing factors. An enhanced residual network and an Encoder–Decoder Model for turbine blade ICT images artifact removal have been designed. Through the analysis and discussion of results, it is found that receptive field and spatial resolution have a great impact on model performance.
- We propose a Dilated Residual Network for turbine blade ICT images artifact removal. We introduce dilated convolution, which can increase the receptive field while maintaining spatial resolution.

## 2. Dataset for Turbine Blade ICT Images Artifacts Removal

This paper adopts the supervised CNN for turbine blade ICT images artifact removal. We need to use the turbine blade CT images artifact removal dataset with labels in the training and validation process, including CT images with artifacts as the training samples and the corresponding CT images without artifacts as labels. The dataset’s quality will seriously affect the trained model’s CT image artifact removal effect.

Since there is no open-source dataset for turbine blade ICT images artifact removal that has been reported in previous works, this paper proposes a dataset preparation method for turbine blade CT images artifact removal, which can also be applied to other industrial CT images.

The boundary between useful information and artifact information, i.e., the blade’s edge, is a continuous smooth curve. Therefore, we can manually mark plenty of points on the blade edge, then connect these points with a smooth curve, and useful information and artifact information can be separated using this curve. After that, the artifact information is filled to zero, and we can obtain the label images without the artifact information.

We adopt the Bézier curve for the multi-point curve fitting, which is a mathematical curve applied to two-dimensional graphics applications. The Bézier curve uses a series of control points to parameterize a continuous smooth curve. It is one of the most important parametric curves in computer graphics. It is defined as

(1) $$\vec{P(t)}=\sum_{i=0}^{n-1}\vec{P_{i}}B_{i,n}\left(t\right),t\in \left[0,1\right]$$

where *i* denotes the index of control points, starting from 0; P(t) denotes the coordinate vector on the curve; $P_{i}$ denotes the coordinate of control points; *n* denotes the number of control points; and $B_{in}\left(t\right)$ denotes the primary function of the Bézier curve.

**Figure 2:**
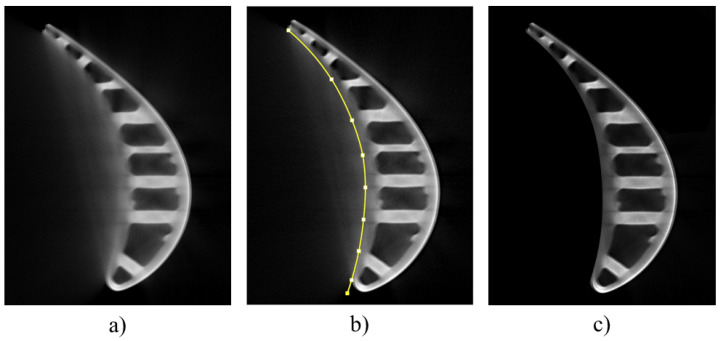
(**a**) The original ICT image; (**b**) the contour fitting; (**c**) the label without artifacts for (**a**).

Figure 2 shows an example of creating a label without artifacts for a training sample. When manually preparing an ICT image (see Figure 2a) to remove artifacts, we fit the contour of the blade first (see Figure 2b) and then set the image outside the contour to zeros to remove artifacts (see Figure 2c).

Given the problem that the amount of original data of ICT images is less, which affects the model’s training, we augment the dataset through rotation, mirroring images randomly, and other data enhancement technologies. Data augmentation is a common way to expand datasets in deep learning. When the dataset is minor, various methods are often used to expand the dataset to prevent overfitting during the model training. There are many ways to augment datasets, and we adopted the following methods:

- Flip: Flip the image horizontally or vertically.
- Scale transformation: Enlarge or reduce the image according to the specified scale factor, or refer to the SIFT feature extraction idea, use the specified scale factor to filter the image to construct the scale space, and change the size or blur degree of the image content.
- Rotation/reflection transformation: Randomly rotate the image at a random angle and change the image’s orientation.
- Shift transformation: Randomly translate the image on the image plane. The translation range and step size can be specified randomly or manually, and the translation can be carried out in the horizontal or vertical direction to change the position of the image content.
- Contrast transformation: In the HSV color space of the image, change the saturation S and V luminance components and keep the hue H unchanged. Each pixel’s S and V components are exponentially calculated (the exponential factor is between 0.25 and 4) to increase the illumination change.

**Figure 3:**
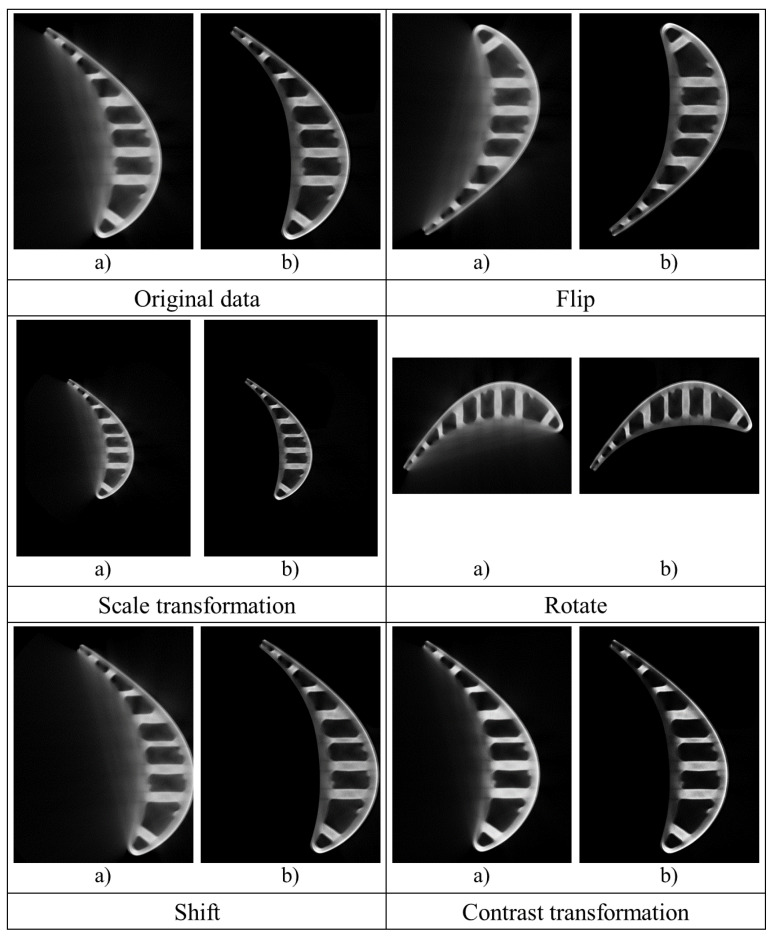
(**a**) The original ICT image; (**b**) the label for (**a**).

Figure 3 shows some examples of data augmentation, where we uniformly apply operations such as flipping, scale transformation, rotating, shifting, and contrast transformation to a training image and its corresponding label. Flipping, rotating, or shifting can simulate the flipping, rotating, or shifting of the target (i.e., blade) itself in the real scenarios; scaling can simulate the size change or forward and backward movement of the target in the real scenarios; and contrast transformation can simulate the different densities of the target in the real scenarios.

## 3. Enhanced Residual Network and Encoder–Decoder Model for Turbine Blade ICT Images Artifact Removal

Since the objective of the artifact removal task is close to that of image restoration, we use the residual network and the Encoder–Decoder models, which are commonly used in image restoration tasks, to study artifact removal and explore the main influencing factors in artifact removal model design.

### 3.1. Enhanced Residual Network for Turbine Blade ICT Images Artifacts Removal

**Figure 4:**
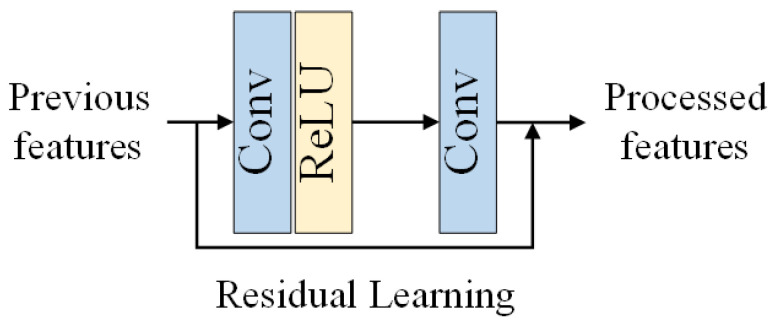
The Enhanced Residual Block (ERB).

**Figure 5:**
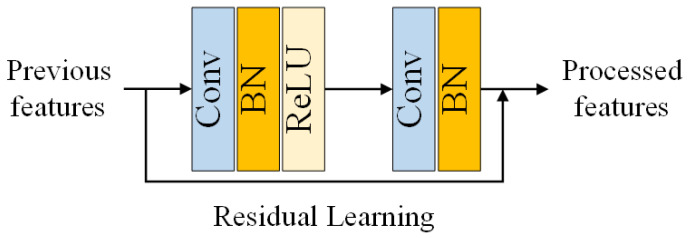
The original Residual Block (RB).

In order to achieve better performance, EDSR [27] proposes the Enhanced Residual Block (ERB, see Figure 4), which removes the Batch Normalization (BN) layers in the original Residual Block (RB, see Figure 5) in ResNet [28]. EDSR [27] found that (1) BN layers have been shown to improve the generalization performance of deep neural networks by normalizing the activations of each layer, which can reduce the internal covariate shift and help prevent overfitting. However, in the context of image restoration, the BN layers did not provide any significant benefits and even slightly degraded the performance of the network. (2) BN layers will introduce additional computational overhead, as they require additional forward and backward passes during training and inference. Removing the BN layers from the RB can reduce the number of operations required and make the network more efficient. (3) EDSR argued that the use of BN layers may not be necessary in the context of image restoration, as the network is able to learn the normalization of the activations on its own through the residual learning mechanism. The removal of BN layers in ERB was motivated by the desire to improve the performance and efficiency of the network for image restoration, without the need for additional normalization layers. We can also verify this conclusion in the results comparison of DnCNN and ERAR in Section 5.2.

Suppose we have previous features $F_{in}$, the blocks can obtain the processed features $F_{out}$ through ERB by

(2) $$F_{out}=Conv\left(ReLU\left(Conv\left(F_{in}\right)\right)\right)+F_{in}$$

where Conv(·) denotes a standard convolutional operation and ReLU(·) denotes the function of the rectified linear unit (ReLU).

**Figure 6:**
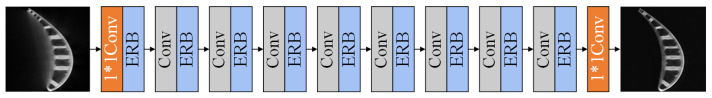
The structure of the enhanced residual network for ICT images artifact removal.

As shown in Figure 6, the proposed enhanced residual network for turbine blade ICT images artifact removal (ERAR) uses a 1×1 Conv and an ERB to extract shallow features, eight convolutional layers followed by eight ERBs to extract hierarchical features, and a 1×1 Conv to reconstruct the ICT images without artifacts.

### 3.2. Encoder–Decoder Model for Turbine Blade ICT Images Artifact Removal

**Figure 7:**
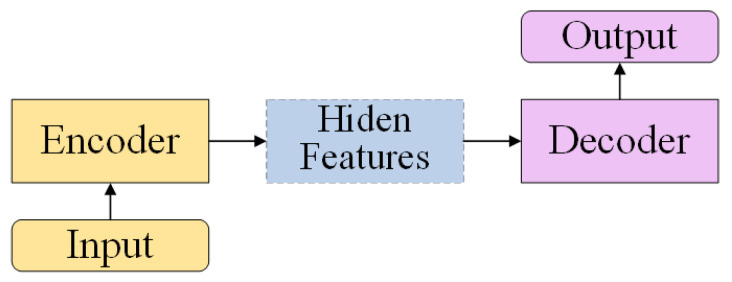
The abstract structure of the Encoder–Decoder framework.

The Encoder–Decoder framework is a general deep learning framework, which is widely used in text translation [29,30], image generation [31,32], and image restoration [33,34]. The Encoder–Decoder model can be abstractly represented as Figure 7. Given the input, the encoder encodes the input nonlinear transformation into hidden features and then decodes the hidden features through the decoder to transform the hidden features into the target output. One advantage of the Encoder–Decoder framework in computer vision is that it allows the efficient processing of high-resolution images by reducing the dimensionality of the input data through the encoder portion of the network. This allows the network to run faster and with less computational resources. Another advantage is that the Encoder–Decoder framework allows for the generation of high-quality output images, as the decoder portion of the network is able to reconstruct the output image using the encoded representation of the input image. This is particularly useful for tasks such as image restoration (e.g., artifact removal), where the quality of the output image is important.

**Figure 8:**
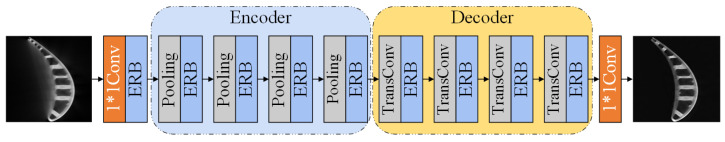
The structure of the Encoder–Decoder model for turbine blade ICT images artifact removal.

Based on the Encoder–Decoder framework, we designed an Encoder–Decoder model for turbine blade ICT images artifact removal (EDAR), as shown in Figure 8. In the same manner as ERAR, EDAR uses an RB and an ERB to extract shallow features. For the encoder, we use four Max Pooling layers for data dimensionality reduction and four ERBs after each Pooling layer for further feature extraction at the scale. For the decoder, we use four transposed convolutional layers to upgrade the dimension of the hidden features to the original resolution and four ERBs after each transposed convolutional layer to refine the features. Finally, we use an RB to reconstruct the ICT images without artifacts.

### 3.3. Analysis and Discussion

We have designed two models for turbine blade ICT Images artifact removal: ERAR and EDAR. They are roughly similar in network structure, and the main difference is the Receptive Field (RF) and the resolution of the feature map. In this section, we analyze the above two characteristics of these two models.

The RF of a neuron in a convolutional neural network (CNN) is a region in the input space that the neuron is sensitive to, i.e., the region of the input that the neuron uses to compute its output. The RF can have a significant impact on the behavior of the CNN and the information it can extract from the input. For a CNN with *N* convolutional layers, an *N*th layer with a kernel size of *k*, and a stride of *s*, the RF of the model can be calculated recursively as follows:

(3) $$RF=RF_{N}+s\times \left(RF_{N-1}-1\right)$$

where $RF_{N}$ refers to the RF of the *N*th layer relative to its previous layer (i.e., N−1th layer), and $RF_{N-1}$ refers to the RF of the N−1th layer relative to its previous layer (i.e., N−2th layer). For any convolutional layer *i*, with a convolution kernel size of *k*, the RF $RF_{i}$ relative to the previous layer can be calculated using the equation:

(4) $$RF_{i}=k$$

***ERAR.*** Because the size of the convolution kernel used in ERAR is small and the step size is 1, the RF of the whole convolution network is small. According to calculation, the RF of ERAR is 35, so the model cannot capture the shape features in the image. It is challenging to recognize the specific area where the artifact occurs. In the feature extraction and processing of ERAR, the resolution of the feature map is always consistent with the input images, which ensures that the output image information will not be lost and the turbine blade in the image can remain clear.

***EDAR.*** The pooling layer also plays a role in expanding RF. In EDAR, the Max Pooling layers with a kernel size of 2 and a step size of 2 are used. After each pooling layer, the RF of the convolution network will increase twice as much as that of the previous layer. According to calculation, the RF of EDAR is 156, which is much larger than EDAR. On the other hand, the pooling layer also reduces the resolution of the feature map, which will lead to the loss of detailed information, and there is little hope of recovering all the details in the subsequent data dimensionality upgrade.

**Figure 9:**
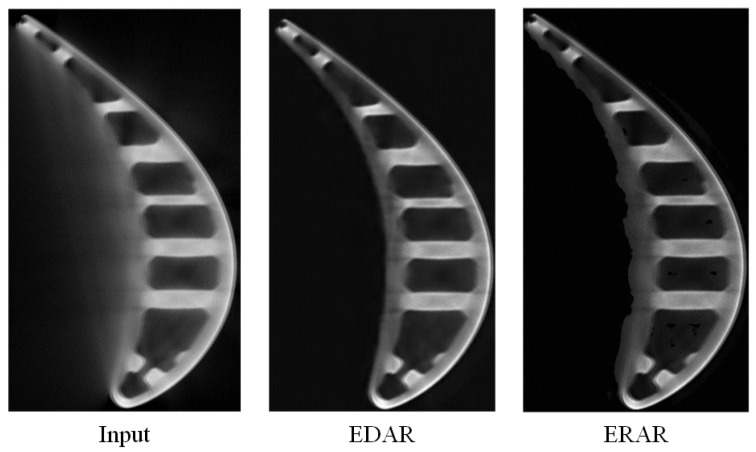
The visual results generated by ERAR and EDAR.

In Figure 9, we compare the visual results generated by ERAR and EDAR. Although EDAR effectively removes artifacts, careful observation of the practical information in the turbine it retains shows that the image becomes blurred and the details are lost in the process of artifact removal. In contrast, the image details in ERAR results are well preserved. However, due to the smaller RF, ERAR cannot capture the shape features in the image and thus accurately judge the turbine area and artifact area in the image, so it cannot judge the contour of the turbine, resulting in a poor artifact removal effect.

## 4. Dilated Residual Network for Turbine Blade ICT Images Artifact Removal

### 4.1. Network Structure

In the previous section, our key idea was to increase the RF of the network while preserving spatial resolution. A simple idea is to remove the subsampling layer (e.g., pooling and striding) in the model, i.e., ERAR, to keep the resolution of the feature map but cause the reduction of the RF. This severely reduces the quality of artifact removal. For this reason, we use dilated convolutions to increase the RF of the model and keep the spatial resolution.

**Figure 10:**
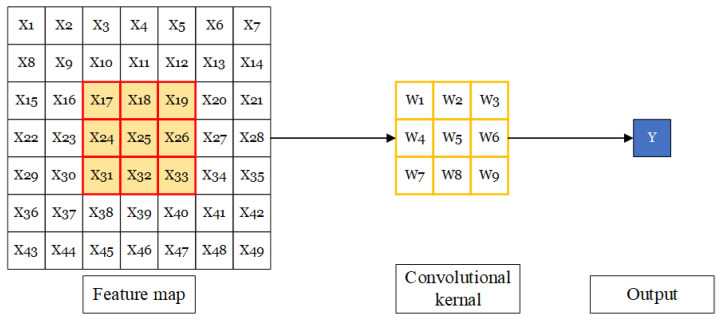
A standard convolutional operation.

A standard convolutional operation is shown in Figure 10. It is the usual sliding window operation, where the elements in the window are always adjacent elements in the input feature map. In Figure 10, we use a convolutional kernel with a size of 3×3 as an example; the RF of this convolutional layer is 3.

**Figure 11:**
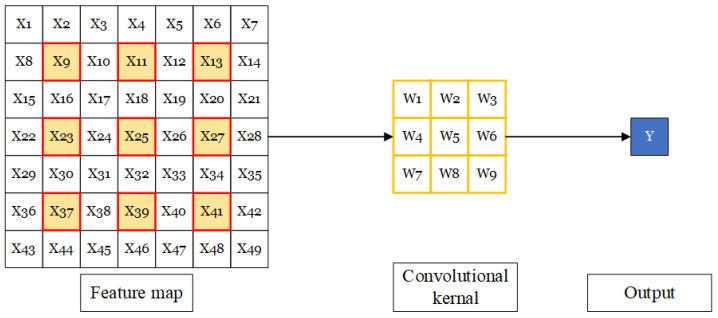
A dilated convolutional operation with a size of 3×3 and a dilation rate of 2.

A dilated convolutional operation is shown in Figure 11. It is equivalent to dilating the filter before doing the usual convolution. Dilating the filter means expanding its size by filling the empty positions with zeros. In dilated convolution, no expanded filter is created; instead, the filter elements are matched to distant (not adjacent) elements in the input matrix. The distance is determined by the dilation rate *d*; generally speaking, the distance is d−1. The RF of dilated convolution will increase as the dilation rate is increased. The number of elements of the filter remains the same, but with the increase in dilation rate, they will achieve more coverage. For a dilated convolutional layer *i*, with a convolution kernel size of *k* and dilation rate of *d*, the RF $RF_{i}$ relative to the previous layer can be calculated using the following equation:

(5) $$RF_{i}=\left(k-1\right)\times d+1$$

In Figure 11, we use a convolutional kernel with a size of 3×3 and a dilation rate of 2 as an example; the RF of this convolutional layer is 5, which is larger than a standard convolution with little increase in computational load.

**Figure 12:**
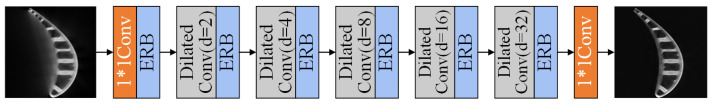
The structure of the Dilated Residual Network for turbine blade ICT images artifact removal.

Specifically, we propose a Dilated Residual Network for turbine blade ICT images artifact removal (DRAR) based on ERAR and EDAR. As shown in Figure 12, DRAR uses ERB as the feature extractor but uses dilated convolutions instead of the standard convolution and pooling layers.

The size of the dilated convolution kernel used in DRAR is 3×3, the stride is 1, and the dilation rate is 2, 4, 8, 16, and 32, respectively. The convolution kernel size in ERB is also 3×3, and the stride is 1. In the data processing process, the feature map’s resolution remains unchanged.

### 4.2. DRAR Analysis

Because DRAR uses dilated convolution, it can significantly increase the RF without changing the resolution of the feature map with little increase in computation load. According to calculation, the RF of EDAR is 156, which is much larger than ERAR and similar to EDAR. Moreover, in the feature extraction and processing of DRAR, the resolution of the feature map is always consistent with the input images, which ensures that the output images information will not be lost and the turbine blade in the image can remain clear.

## 5. Experiments and Results

### 5.1. Experiments Settings

***Implementation details.*** In ERAR, EDAR, and DRAR, we set 3×3 as the kernel size of all convolutional layers except those that are specifically designated as 1∗1 convolutional layers. For the convolutional layer with 3×3 kernel size, we pad zeros to each side of the input to keep the feature size fixed, and all convolutional layers have 64 channels. The final output layer has three output channels.

The models are optimized with the $L_{1}$ loss function; given an input image $I_{in}$ and the label image $I_{label}$, our goal is to learn a mapping function $H_{model}\left(\cdot \right)$ for generating an artifact-removed image $I_{AR}=H_{model}\left(I_{in}\right)$ that is as similar to $I_{label}$ as possible. The $L_{1}$ loss function is defined as

(6) $$L_{1}\left(I_{AR},I_{label}\right)=\frac{1}{hwc}\sum_{l,m,n}\left|I_{AR}^{l,m,n}-I_{label}^{l,m,n}\right|$$

where *h* is the height of the label image, *w* is the width of the label image, *c* is the number of channels of the label image, $I_{AR}^{l,m,n}$ is the artifact-removed individual pixel value at row *l*, column *m* and channel *n*, and $I_{label}^{l,m,n}$ is the ground truth individual pixel value.

***Datasets and metrics.*** We prepare a dataset containing 680 ICT images of a turbine blade to train and test our model. The 680 layers were obtained from the reconstruction of one blade, and the details of the acquisition and reconstruction process are shown in Table 1. The resolution of original images is 1359×1359.

We used 500 of them for model training, 100 for model validation, and 80 for model testing. The results were evaluated with the Peak Signal-to-Noise Ratio (PSNR) and the Structure Similarity Index Measurement (SSIM) to show the artifact removal effect.

***Training settings.*** We randomly flipped horizontally or vertically and rotated $90^{\circ }$ the images for data augmentation in the training phase. We used Pytorch [35] to implement our models and trained them with the ADAM optimizer with $\beta_{1}=0.9$, $\beta_{2}=0.999$, and $\varepsilon =10^{-8}$. The initial learning rate was $10^{-4}$ and decreased to half every 200 epochs. The batch size was set to 32. We used the GPU of the Nvidia GeForce RTX 2080Ti to train our models. Training proceeded for 500 epochs total, and it took about 6 hours to train a DRAR from scratch.

### 5.2. Results

In this section, we demonstrate the artifact removal performance of DRAR by presenting quantitative and visual results. We present the comparison of DRAR with other deep learning-based methods including ERAR and EDAR, as mentioned in this paper, as well as MFCNN [24], UNet [22], and DnCNN [36] (a method for image denoising) from previous work. It is worth noting that while DnCNN is specifically designed for image denoising, the artifacts in ICT images can also be considered as noises, and so we also compare DnCNN with DRAR. We trained all the mentioned methods using the dataset in this paper and compared their results.

**Figure 13:**
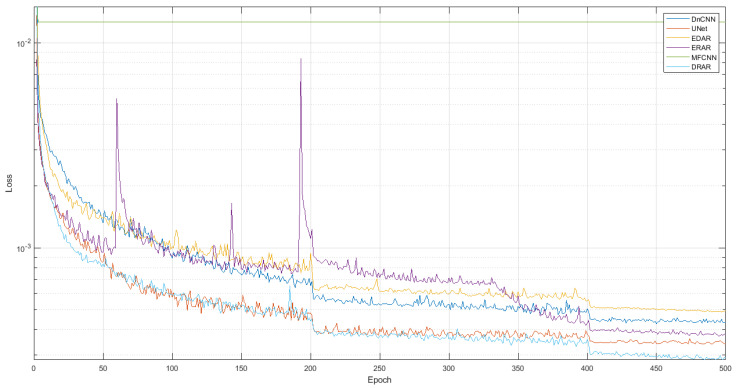
Loss value of DnCNN, ERAR, MFCNN, UNet, EDAR, and DRAR during training process.

**Figure 14:**
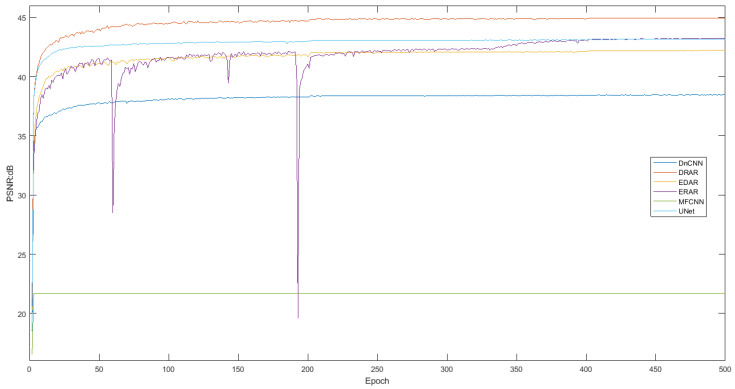
PSNR of DnCNN, ERAR, MFCNN, UNet, EDAR, and DRAR during training process.

***Training process.*** We show the performance of each model on the training set and the validation set during the training process. For the training set, the loss value of each model on the data is shown (see Figure 13), and for the validation set, the PSNR of each model on the data is shown (see Figure 14).

From those results, we can see that MFCNN cannot provide converge for the data mentioned in this paper (the reason is speculated to be that the number of layers in MFCNN is too small, making it difficult to learn the complex non-linear mapping between samples and labels), while the other networks can converge well, and DRAR can achieve a smaller loss value (better) and a higher PSNR (better).

***Quantitative results.***Table 2 shows the quantitative results of DnCNN, ERAR, MFCNN, UNet, EDAR, and DRAR. In general, DRAR achieves superior performance. For PSNR and SSIM indicators, with less computational load and processing time, all of the results of DRAR are the best. We notice that UNet and EDAR perform poorly, which is mainly because the resolution of the feature map is reduced in the process of data processing; then, the image becomes blurred due to the inability to recover all the details in the process of data dimensionally upgrading, resulting in low scores. In contrast, DRAR takes into account the advantages of a large RF and constant spatial resolution, so it achieves the best performance in all indicators.

**Figure 15:**
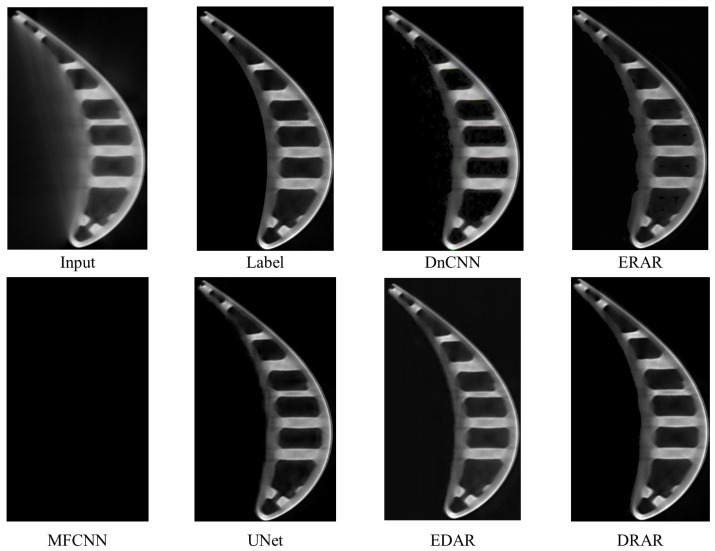
Visual results of DnCNN, ERAR, MFCNN, UNet, EDAR, and DRAR; the results of MFCNN are all zeros. Enlarged for best viewing.

***Visual results.*** In Figure 15, we compare the visual results generated by DnCNN, ERAR, MFCNN, UNet, EDAR, and DRAR, where MFCNN is unable to converge and the results of MFCNN are all zeros. Since DnCNN and ERAR keep the resolution unchanged during image processing, their results retain better image details. However, due to the smaller RF, ERAR cannot capture the shape features in the image and thus accurately judge the turbine area and artifact area in the image, so it cannot judge the contour of the turbine, resulting in a poor artifact removal effect. In contrast, UNet and EDAR are able to effectively remove artifacts; however, it can be seen from the generated blade images that the results become blurry and the details are lost in the process of removing artifacts. While DRAR has a larger RF and maintains spatial resolution, its processing results effectively remove artifacts, and the turbine in the image is precise and sharp. Such an obvious comparison proves that DRAR has a more robust ability to combine and represent features, which can produce sharper and more convincing results from LR images.

***The effect of data augmentation.*** We conduct control variable experiments to analyze the effect of data augmentation. We set the DRAR that used data augmentation during training as the baseline, referred to as “DRAR with augment”. Then, we re-trained a DRAR without using data augmentation following the same experiment setting as in Section 5.2, referred to as “DRAR w/o augment”, and compared and analyzed the effect of data augmentation through the results generated by them.

**Figure 16:**
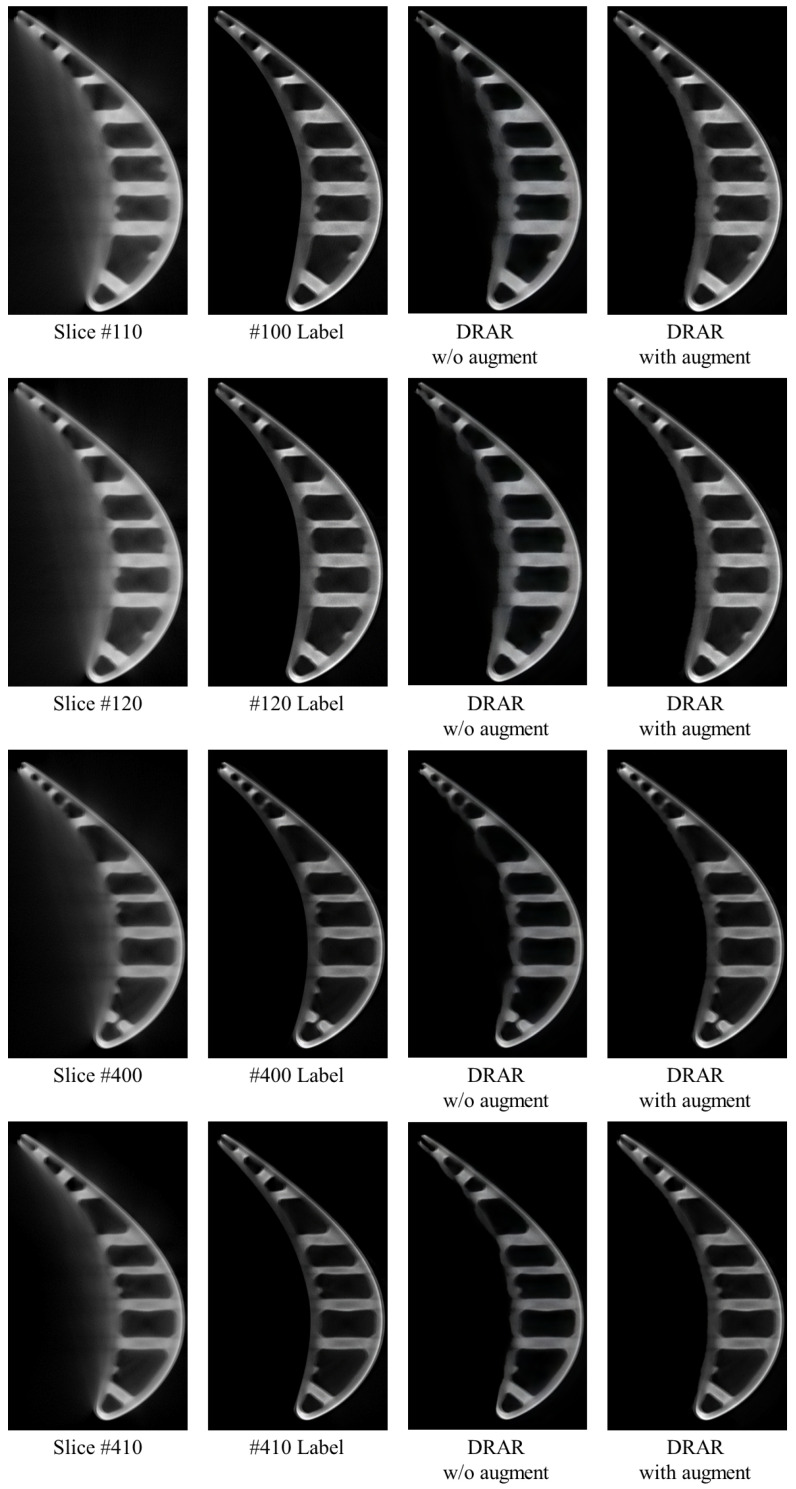
Visual results of DRAR w/o augment and DRAR with augment. Slices #110, #120, #400, and #410 are shown from top to bottom, where the impact of artifacts is more severe in slices #110 and #120, and relatively mild in slices #400 and #410. Enlarged for best viewing.

Table 3 shows the quantitative results of DRAR w/o augment and DRAR with augment. Due to the small number of samples (a total of only 640 images are used to prepare the dataset), the DRAR w/o augment overfits to the existing data. In other words, the DRAR w/o augment learns to memorize the location or direction where artifacts occur and directly treat this area as artifacts and remove them (this can be seen in rows 3 and 4 of Figure 16, where DRAR w/o augment incorrectly removes parts of the blade as artifacts in slices #400 and #410.). By data augmentation, the inherent symmetry of the ICT image is broken, but the number of images and the diversity of the dataset are increased; in addition, the location and direction where the artifacts occur are not fixed, which will force DRAR with augment to learn how to recognize the pattern of artifacts and achieve more accurate artifact removal.

## 6. Conclusions

This paper proposes a dilated residual network for industrial computed tomography image artifact removal (DRAR). Firstly, we designed an enhanced residual network for turbine blade ICT image artifact removal(ERAR) and an Encoder-Decoder model for turbine blade ICT image artifact removal(EDAR). By comparing and analyzing their performance and characteristics, we found that the network’s receptive field and spatial resolution are crucial factors in artifact removal for ICT images. Based on this observation, we designed DRAR with dilated convolution and enhanced residual block, which utilizes dilated convolution with varying dilation rates. DRAR exhibits the characteristic of a large receptive field while maintaining spatial resolution. Extensive experiments demonstrated that DRAR is effective in achieving both quantitative and visual improvements.

## Figures and Tables

**Table 1:** The details of the acquisition and reconstruction process.

Acquation	beam geometry	cone-beam
	mAs	0.26 mA × 1 s
	kVp	300
	angle	24
Reconstruction	algorithm	FDK
	filter	3 mm tin filter board
	correction	None

**Table 2:** Quantitative results comparison, the best results are **highlited**. DRAR achieves the best performance. The computational load and processing time are evaluated on an image with a resolution of 512×512.

Model	RF	Resolution of Feature Map	PSNR:dB	SSIM	Computational Load: GFlops	Processing Time: ms
DnCNN	35	Preserving resolution	38.46	0.9325	392.1	191.9
ERAR	35		43.24	0.9741	388.4	146.6
MFCNN	68	Downsampling before upsampling	–	–	49.8	21.7
Unet	160		43.16	0.9710	458.7	60.42
EDAR	156		42.21	0.9687	437.2	59.81
DRAR	149	Preserving resolution	**44.95**	**0.9806**	**328.8**	**38.19**

**Table 3:** Quantitative results of DRAR w/o augment and DRAR with augment. We report the best PSNR on the test set.

Model	Data Processing	PSNR: dB	SSIM
DRAR	without Data Augmentation	39.08	0.9659
	with Data Augmentation	44.95	0.9806

## Data Availability

The data presented in this study are available on request from the corresponding author. The data are not publicly available due to confidentiality.
